# Adaptive Control of the Redundant Axis of a Surgical Robot for Operating Room Workspace Optimization Using Reinforcement Learning

**DOI:** 10.3390/s26092881

**Published:** 2026-05-05

**Authors:** Irati Renedo-Alonso, Juan A. Sánchez-Margallo, Nestor Arana-Arexolaleiba, Íñigo Elguea-Aguinaco

**Affiliations:** 1 Robotics and Automation Electronics and Computer Science Department, University of Mondragon, 20500 Mondragon, Spain; irati.renedo@alumni.mondragon.edu (I.R.-A.); narana@mondragon.edu (N.A.-A.); 2 Bioengineering and Health Technologies Unit, Jesús Usón Minimally Invasive Surgery Centre, 10071 Cáceres, Spain; jasanchez@ccmijesususon.com; 3 Research & Development Department, Electrotécnica Alavesa S.L., 01010 Vitoria-Gasteiz, Spain; 4 Research & Development Department, Sortech 2030 S.L., 31800 Alsasua, Spain

**Keywords:** fuzzy logic, human–robot interaction, laparoscopy, reinforcement learning, surgical robotics

## Abstract

Laparoscopy is one of the most widely used surgical techniques in clinical practice. However, its practice is associated with medium- and long-term musculoskeletal disorders in surgeons. In this context, robot-assisted surgery has emerged as a promising approach for mitigating ergonomic constraints while enhancing control and precision during laparoscope manipulation. Despite these advances, existing research predominantly focuses on robotic control strategies, whereas the study of human–robot interaction in the operating room remains comparatively underexplored. This paper presents a proof-of-concept framework for workspace-aware posture adaptation in collaborative surgical robotics. The proposed approach combines vision-based human activity recognition with reinforcement learning to control the shoulder–elbow–wrist redundant angle of a seven-degree-of-freedom manipulator holding a laparoscope. Based on the detected interaction context, the system distinguishes between controlling, observing, cutting, and blocked states. During the observation and cutting phases, the controller allows the robot’s posture to be reconfigured so that it tilts away from the human operator while maintaining the position of the laparoscope; when the surgeon moves away, the robot gradually returns to its default configuration. Two reward formulations, dense and fuzzy, are compared. Real-world experiments show that both approaches learn the desired reflexive behavior, while the fuzzy reward yields improved training stability and more consistent real-system performance, increasing workspace availability around the surgeon.

## 1. Introduction

Laparoscopy is one of the most widely adopted minimally invasive surgical techniques in modern clinical practice, offering significant advantages over open surgery, including reduced tissue trauma, lower postoperative pain, shorter hospital stays, and faster recovery [1,2,3]. These benefits have led to widespread adoption across multiple specialties, such as general surgery, gynecology, urology, and oncology. Despite its clinical success, laparoscopic surgery imposes substantial physical and cognitive demands on surgeons due to constrained workspaces, indirect visualization through monitors, limited instrument dexterity, and prolonged static postures [4,5,6].

Indeed, numerous studies have reported a high prevalence of work-related musculoskeletal disorders among laparoscopic surgeons, affecting the neck, shoulders, back, wrists, and hands [7,8]. These issues not only compromise surgeon well-being but can also negatively impact procedural efficiency, precision, and patient safety [6,8].

Accordingly, robot-assisted surgery has emerged as a promising solution to mitigate some of these limitations by improving instrument precision, motion stability, and ergonomic comfort [9,10]. Robots can relieve surgeons from physically demanding tasks such as manipulation of the laparoscope. However, the majority of robotic systems rely on explicit user commands (e.g., foot pedals, voice control, or manual interfaces) and operate under predefined control strategies [11,12]. As a result, they exhibit limited awareness of the surgeon’s posture, activity, and spatial relationship with the robot, which restricts their ability to adapt dynamically to the clinical environment.

**Figure 1:**
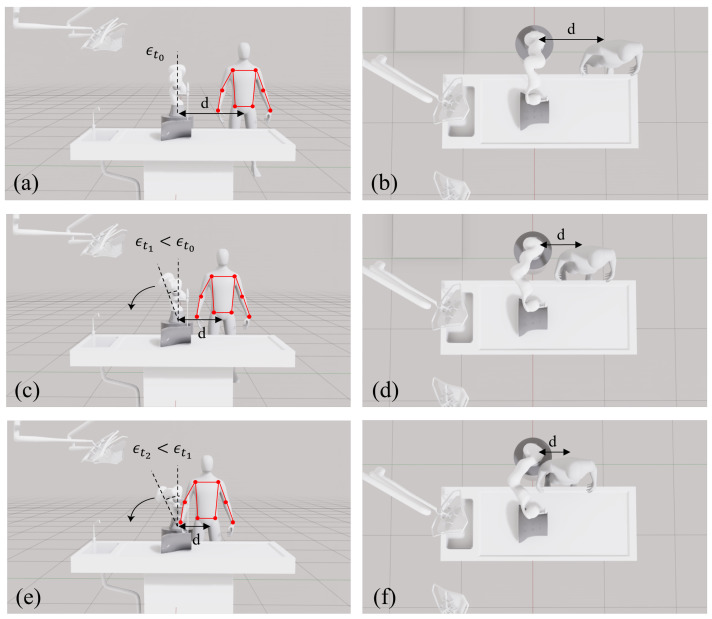
SEW redundant angle reconfiguration sequence for workspace-aware posture adaptation. The variable *d* denotes the lateral distance between the human operator and the robot, while ϵ represents the SEW redundant angle defining the arm posture. In (**a**,**b**), the human remains at a safe distance and the manipulator maintains its nominal configuration. In (**c**,**d**), as the human approaches the robot (decreasing *d*), the redundant SEW angle is progressively adjusted, tilting the arm away from the human ($\epsilon_{t_{1}}<\epsilon_{t_{0}}$) while preserving the laparoscope pose. In (**e**,**f**), at close proximity, the robot reaches its maximum tilt ($\epsilon_{t_{2}}<\epsilon_{t_{1}}$), increasing the available workspace around the human operator. Arrows indicate the direction of human approach and the corresponding posture adaptation of the robot.

In scenarios where the surgeon works close to a robot, static robot configurations can lead to reduced available workspace, awkward postures, or even potential collisions. In particular, redundant manipulators such as the KUKA LBR iiwa (KUKA AG; Augsburg, Germany) provide additional degrees of freedom that can be exploited to reconfigure the robot posture without affecting the laparoscope pose. This redundancy can be parameterized through the shoulder–elbow–wrist (SEW) angle, which describes the swivel motion of the arm about the shoulder–wrist line and allows multiple joint configurations to achieve the same tool pose [13]. In this paper, this redundant degree of freedom is referred to interchangeably as the SEW angle or the redundant axis. By adjusting this SEW angle, the robot can tilt away from the surgeon while maintaining a fixed laparoscope position and orientation, as shown in Figure 1.

Conventional redundancy resolution approaches, including null-space optimization or artificial potential field (APF)-based strategies, can achieve posture adaptation but often require carefully tuned objective functions and may struggle to react robustly under unpredictable human motion. In addition, APFs formulations may suffer from local minima when attractive and repulsive forces become similar [14].

In this sense, reinforcement learning (RL) methods emerge as a promising approach, as they hold the promise of solving control tasks in complex, unstructured environments. Indeed, they allow agents to learn through interaction with their surroundings and, ideally, to generalize the learned behavior to new, unseen scenarios [15]. Unlike model-based control methods, RL does not require an explicit analytical model of the system dynamics and can learn policies that balance multiple objectives under uncertainty [16,17]. This makes RL particularly suitable for human–robot interaction (HRI). Moreover, this adaptability in dynamic and uncertain settings may be further strengthened when combined with fuzzy rewards [18], as they enable smooth and continuous reward shaping through soft transitions between states, allowing the reward structure to adapt to the interaction context, reducing sensitivity to noise and abrupt changes in the reward landscape. However, while RL has been successfully applied to surgical instrument manipulation and autonomous task execution, its use for HRI and workspace adaptation in the operating room remains underexplored [19,20].

In this work, we propose a proof-of-concept framework that combines computer vision-based human activity recognition with RL to adaptively control the redundant axis of a collaborative surgical robot. First, we recognize three meaningful human activities in the operating room, namely, *controlling*, *observing*, and *cutting*. Additionally, a safety state denoted as *blocked* is defined to represent situations in which the spatial configuration of detected individuals may compromise safe HRI. This activity recognition and spatial position are inferred in real time using a vision-based perception system, enabling the robot to become aware of the operative context. An RL agent is then used to control the redundant degree of freedom of a seven-degree-of-freedom robotic manipulator, allowing the robot to tilt away from the surgeon or nurse when proximity increases while preserving the laparoscope pose. Furthermore, when the distance to the surgeon or nurse increases, the robot smoothly returns to a nominal configuration, maintaining functional efficiency and spatial balance. The RL agent is trained and evaluated using two reward formulations, traditional dense and fuzzy, enabling a comparative analysis of performance and robustness. The present study focuses on the methodological control architecture and its feasibility for HRI-oriented workspace adaptation, while ergonomic assessment, safety validation, and perceived-safety evaluation remain as future work.

Therefore, the main contributions of this paper can be summarized as follows:

- A vision-based human activity recognition framework is introduced to infer surgeon or nurse activity and proximity in real time, enabling perception-driven and context-conditioned robot behavior without requiring additional explicit user commands.
- A perception-conditioned RL controller is proposed to regulate the SEW redundant degree of freedom of a collaborative laparoscope-holding manipulator, allowing proactive and context-aware posture reconfiguration to increase workspace clearance while preserving the laparoscope pose.
- Two reward formulations, traditional dense and fuzzy, are investigated, where the fuzzy formulation introduces a context-dependent reward shaping strategy that enables smooth and continuous adaptation of the tracking penalty as a function of HRI, providing a comparative analysis of training stability and robustness for redundancy adaptation under uncertain human motion.
- The complete hierarchical perception–decision–control architecture is implemented and validated as a proof-of-concept on a real KUKA LBR iiwa platform in a realistic laboratory setup, demonstrating the feasibility of integrating high-level semantic perception with learning-based redundancy adaption for HRI-oriented workspace-aware posture adaptation.

This paper is structured as follows. Section 2 reviews related works on RL in surgical robotics and HRI in operating rooms. Section 3 describes the proposed methodology, including the overall system architecture, activity recognition pipeline, and RL formulation. Section 4 presents experimental results, while Section 5 discusses the implications and limitations of the proposed approach. Finally, Section 6 concludes the paper and outlines future research directions.

## 2. Related Works

### 2.1. Reinforcement Learning in Surgical Robotics

In recent years, RL has attracted interest in the field of surgical robotics [21]. Unlike classical control and planning approaches, RL does not require an explicit analytical model of the system dynamics and can handle stochasticity and high-dimensional state spaces, which makes it appealing for surgical settings where tissue properties, tool–tissue interaction, and intraoperative conditions are difficult to model accurately.

Most applications of RL in surgical robotics have focused on autonomous or semi-autonomous execution of surgical subtasks, such as instrument positioning, cutting, suturing, and manipulation of deformable tissues. In particular, tensioning and cutting of soft tissues constitute representative challenges due to the highly nonlinear behavior of deformable materials and the need to continuously adapt cutting forces as the incision progresses [22].

In this sense, Thananjeyan et al. [23] trained an RL agent for two-dimensional surgical tensioning and cutting using a finite-element tissue simulator, achieving accurate and sensitive cutting performance. However, their study highlighted persistent simulation-to-reality discrepancies, which occasionally resulted in entanglement and undesired tissue deformation during real executions. Moreover, the policy relied on a fixed pinch point throughout the procedure, limiting its applicability to more complex cutting patterns. Ou and Tavakoli [24] partially addressed these limitations by introducing a sim-to-real framework for internal tissue point manipulation that utilizes Bayesian optimization for preoperative planning of optimal grasping points to minimize local tissue deformation.

Building upon these works, Nguyen et al. [25,26] proposed autonomous multi-pinch-point tension planning strategies to improve cutting accuracy, while their results demonstrated enhanced performance, the tensioning directions were constrained to a small discrete set, which may restrict scalability to realistic three-dimensional surgical scenarios. Expanding on this, He et al. [27] introduced an intuition-guided RL framework that coordinates simple intuitive pulling with deliberate RL-based strategies. To overcome the constraints of fixed pinch points, their approach utilizes an autonomous grasp point selection network to ensure targets remain reachable in intricate in vivo environments while avoiding unknown obstacles and lesions.

Other studies have investigated approaches to improve sample efficiency and feature selection through human demonstrations. Shin et al. [28], for instance, combined model-based RL with learning from demonstration to better capture soft-tissue manipulation dynamics. Similarly, Krishnan et al. [29] segmented gauze tensioning and cutting tasks on a da Vinci^TM^ (Intuitive Surgical; Sunnyvale, CA, USA) platform into shorter subtasks with local reward functions to facilitate convergence. Pedram et al. [30] incorporated human knowledge into approximate Q-learning by selecting simplified but informative tissue manipulation features, enabling the agent to learn effective control actions. Nevertheless, these demonstration-based methods often face poor exploration, requiring extensive data to solve tasks without prior knowledge. Huang et al. [31] addressed this by proposing a demonstration-guided exploration approach, which improved sample efficiency by regularizing both the actor and the critic with expert-like behaviors, effectively mitigating the value misestimate issues that often hinder exploration in complex robotic tasks. Nonetheless, these works consistently emphasize the need to extend current methods toward less constrained three-dimensional action spaces with higher dimensionality and stronger generalization capabilities.

More recent studies have directly targeted these requirements by moving into contact-rich three-dimensional environments. Fan et al. [32], for instance, proposed an approach to integrate safety requirements into three-dimensional tasks like debridement. By projecting unsafe actions onto the tangent space of a learned constraint geometry, their framework ensures that the robot bypasses blood vessels and healthy tissue without requiring a prior model of the constraints. Addressing the challenge of higher dimensionality and real-time responsiveness in dynamic three-dimensional settings, Leng et al. [33] developed a method for flexible needle puncturing. Their approach handled harmonic organ motion, such as respiratory displacement, by modeling movement and using Prioritized Experience Replay (PER) to focus the agent’s learning on critical, high-impact surgical events.

However, like many contemporary RL methods, these approaches typically treat surgical tasks in isolation, requiring independent training from scratch for each new procedure and limiting the transferability of learned skills. To address this procedural bottleneck, Ho et al. [34] proposed the SurgIRL paradigm for life-long learning and generalization. Through this approach, the authors enabled surgical robots to accumulate and reuse heterogeneous policies across diverse sequences of tasks, allowing the system to build expertise over time rather than learning each new skill independently.

Beyond direct surgical manipulation, RL has also been explored in the optimization of clinical decision-making processes, including anesthesia delivery, drug dosing, and therapy personalization [19,20,35]. In such applications, agents learn policies that balance competing objectives, such as therapeutic effectiveness and safety constraints, highlighting the broader potential of RL for adaptive decision-making in healthcare systems.

Despite these advances, the majority of RL-based approaches in surgical robotics remain primarily task-oriented, focusing on improving manipulation accuracy, autonomy, or procedural efficiency. Comparatively, limited attention has been devoted to workspace-aware or human-centered control strategies that dynamically adapt robot configuration based on the surgeon’s or nurse’s posture, spatial location, or activity.

### 2.2. Human–Robot Interaction in Operating Rooms

In the operating room, HRI plays an important role in the development of robotic assistance systems, with particular emphasis on safety, usability, and intuitive control interfaces [36,37].

Early robotic camera holders and laparoscope assistants relied on explicit user inputs, such as foot pedals, voice commands, or head tracking, to control camera orientation and positioning [11]; while these interfaces reduce the need for manual manipulation, they often introduce additional cognitive load and interrupt the natural workflow of the surgeon [38].

More recent approaches have explored context-aware robotic systems capable of responding to surgeon motion or intent. Vision-based methods have been proposed to track surgeon posture and gestures, enabling more intuitive robot responses and reducing reliance on explicit commands [39]. Similarly, human activity recognition techniques have been investigated to identify surgical phases or actions, supporting higher-level assistance and coordination in robot-assisted procedures.

However, most HRI studies in the operating room continue to rely on rule-based systems or supervised learning methods to interpret human behavior [39]. These approaches require predefined thresholds, handcrafted features, or extensive labeled datasets, which can limit their robustness and adaptability in dynamic surgical environments. Moreover, robot behavior is often governed by fixed control policies that do not improve through interaction experience.

Relevant efforts have nevertheless explored workspace-aware collaborative behavior in teleoperated minimally invasive surgery through redundancy exploitation. In particular, Su et al. [40] proposed a safety-enhanced HRI controller for a seven-degrees-of-freedom redundant surgical manipulator, where the null-space swivel motion was used to provide flexible workspace for nurses and staff while a safety constraint limited this compliant motion near kinematic limits or potential collisions. However, the interaction was treated primarily through physical compliance and disturbance compensation rather than through explicit perception of human activity or spatial context.

This line of research was later extended in an improved hierarchical collaborative control scheme, also by Su et al. [41], where the surgical task, the remote center of motion (RCM) constraint, and the compliant swivel behavior were decoupled through a hierarchical operational-space formulation. In that work, redundancy was exploited in multiple null spaces: one to maintain the RCM constraint and another to regulate compliant swivel motion, while a decoupled adaptive fuzzy compensator improved both tip accuracy and RCM preservation under physical interaction. Although these studies are highly relevant because they explicitly exploit redundancy to create flexible workspace around the surgical robot, their HRI model remains mainly force- and constraint-driven, focusing on compliant reaction to physical interaction rather than on proactive, perception-driven adaptation based on detected surgeon or nurse activity and proximity. In [42], the authors argued that addressing these limitations requires a shift toward a more integrated HRI framework, in which healthcare professional-oriented aspects, such as communication and trust, are jointly considered with system-level properties, including learning capability and adaptability. Within this perspective, advances in artificial intelligence and RL are identified as key enablers for moving beyond fixed control policies. Such approaches would allow robotic systems to adapt proactively to dynamic clinical environments and to the specific behaviors and preferences of individual surgeons through continuous, experience-driven optimization.

In parallel, and motivated by the need for more adaptive and context-aware interaction mechanisms, research in industrial robotics has demonstrated the effectiveness of RL for modeling physical HRI, intention inference, and adaptive collaboration. RL-based frameworks have been successfully applied to shared assembly and disassembly tasks, cooperative manipulation, and human-centered control, enabling robots to continuously adapt their behavior based on proximity, motion, and interaction forces [43,44,45,46]. These results suggest that RL can provide a powerful mechanism for learning adaptive HRI strategies in environments characterized by uncertainty.

Despite this progress, the transfer of RL-based HRI approaches to surgical environments remains limited. Unlike industrial settings, surgical environments impose strict workflow constraints, making adaptive HRI particularly challenging. In particular, while redundancy-based workspace adaptation has been explored through compliant and constraint-driven strategies, the use of RL to enable perception-driven, context-aware posture adaptation based on real-time surgeon or nurse activity and proximity in collaborative surgical robots remains largely unexplored.

### 2.3. Research Gap

The literature review highlights two complementary research trends: the growing adoption of RL in surgical robotics for task execution and optimization, and the increasing interest in HRI to improve user experience, safety, and adaptability in the operating room. However, these two lines of research have largely evolved independently.

On the one hand, RL-based surgical robotics research predominantly targets autonomy and manipulation performance, with limited consideration of surgeon ergonomics or spatial comfort. On the other hand, HRI research in the operating room focuses mainly on interface design and perception, often relying on rule-based or supervised methods that lack long-term adaptability and experience-driven optimization.

To the authors’ knowledge, no prior work has explicitly investigated the use of RL to adapt the configuration of a collaborative surgical robot in laparoscopic assistance based on real-time perception of surgeon or nurse activity and proximity, with the explicit goal of optimizing workspace allocation in shared operating-room environments.

Leveraging this use case, we ground the present research on the following hypothesis: *“integrating a fuzzy logic-based reward formulation into the RL framework improves robustness and adaptability in learning compared to a traditional dense reward function, thereby favoring workspace-aware posture reconfiguration under uncertain and unpredictable human motion”*.

To address this gap and evaluate the formulated hypothesis, this work proposes a proof-of-concept that integrates vision-based human activity recognition with RL-based control of the robot’s redundant axis. In particular, the RL agent is trained and compared under two reward formulations, a traditional dense reward, and a fuzzy logic-based reward, in order to assess their impact on robustness and adaptability for workspace-aware posture reconfiguration. By enabling the robot to adapt its configuration dynamically in response to human behavior, the proposed approach aims to improve workspace management, and support more natural HRI in robot-assisted laparoscopy.

## 3. Method

### 3.1. Control Overview

**Figure 2:**
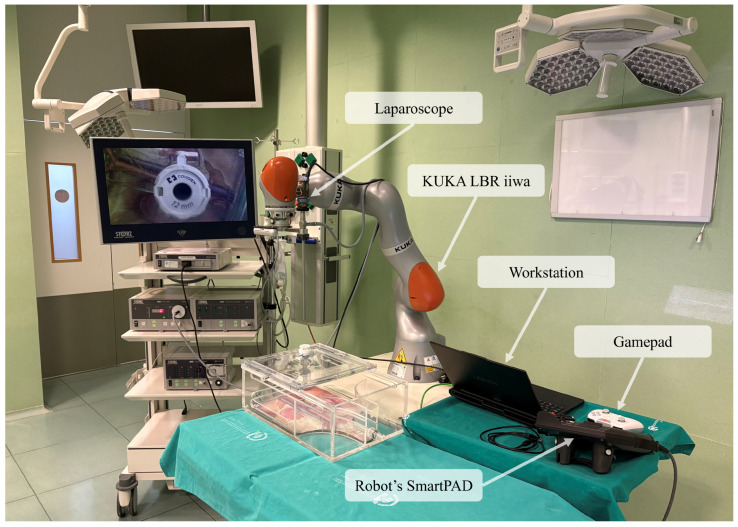
Experimental setup of the experimental operating room environment. A KUKA LBR iiwa collaborative manipulator is equipped with a laparoscope and operates over a laparoscopic box trainer. The system integrates vision-based perception, a workstation for RL and control, and a gamepad interface for manual operation.

The proposed proof-of-concept is implemented in an experimental operating room that emulates the spatial constraints and interaction patterns of an actual robot-assisted laparoscopic operating room. A seven-degree-of-freedom collaborative manipulator KUKA LBR iiwa is employed to hold and orient a laparoscopic camera (KARL STORZ SE & Co. KG; Tuttlingen, Germany), acting as a passive assistant rather than an autonomous surgical actor (see Figure 2).

The control objective is to regulate the redundant joint of the robot while maintaining the laparoscope pose required for visualization. Specifically, the laparoscope pose is preserved, whereas the SEW redundant angle is adjusted to reconfigure the overall arm posture. Before pivoting motions are executed, a point on the laparoscope shaft located approximately at the abdominal wall (cannula) level is recorded and reassigned as the active TCP after complete insertion, replacing the laparoscope tip. This enables subsequent camera motions to be generated with respect to the insertion region, effectively producing pivot-centered motion around this point. The RL controller does not regulate this pivot definition; once this motion scheme is established, it only modulates the redundant SEW swivel motion. In practice, each RL action updates the SEW redundancy parameter while keeping the Cartesian pose of the active motion frame fixed, so the adaptation changes the arm posture without altering the laparoscope viewpoint. This corresponds to a pose-preserving redundancy motion, in which the RL policy affects only the internal configuration of the manipulator without modifying the task space. Since the laparoscope pose remains invariant, the shaft direction is preserved, and the pivot point is consequently maintained in practice. By exploiting kinematic redundancy, the robot can increase workspace clearance around the surgeon during close-proximity interaction without affecting the camera viewpoint or compromising task-imposed constraints such as the RCM.

**Figure 3:**
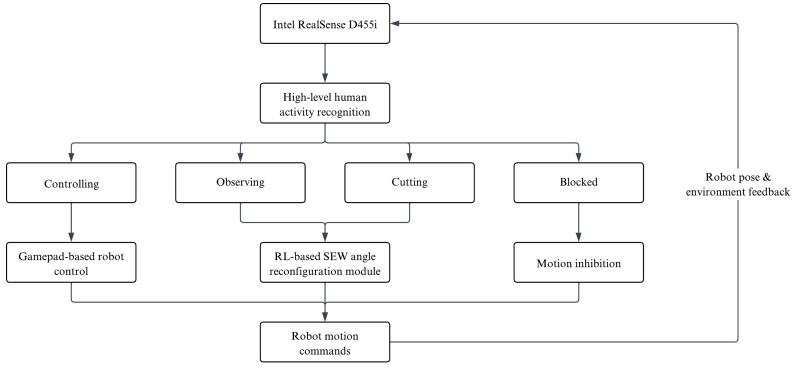
Overview of the proposed control architecture. RGB-D perception feeds a human activity recognition module that infers four interaction states: *controlling*, *observing*, *cutting*, and the safety state *blocked*. These states gate the activation of the control modules. Manual gamepad control is used during the *controlling* state, whereas the RL-based SEW reconfiguration module is activated during *observing* and *cutting* phases. Detection of the *blocked* state triggers a motion inhibition mechanism that suppresses autonomous commands to ensure safe HRI.

Figure 3 provides an overview of the complete perception-to-control pipeline. The architecture follows a hierarchical structure composed of three coupled layers: context perception, interaction-aware decision-making, and low-level robot execution. For clarity and reproducibility, Algorithm 1 summarizes the interaction between these layers.

At the perception level, an external RGB-D camera (Intel RealSense D455i, Intel Corporation; Santa Clara, CA, USA) continuously monitors the laboratory and provides spatial information about the environment. A high-level human activity recognition module processes the visual stream and infers discrete human activity states, namely *controlling*, *observing*, and *cutting*. In addition, a safety state denoted as *blocked* is identified when two individuals are detected on opposite sides of the robot within the interaction area, since SEW axis reconfiguration in this layout could lead to potentially unsafe HRI. These activity estimates serve as contextual triggers that determine whether autonomous redundancy adaption is permitted.

At the decision-making level, the inferred activity is used to gate the activation of the learning-based controller. When the surgeon is in the *controlling* state, the robot is operated directly through a gamepad-based interface that provides standard laparoscope assistance functionalities, including Cartesian control of position and orientation, enforcement of safety boundaries, RCM pivoting, and vision-based alignment with the trocar axis. In this mode, the user retains full authority over the robot motion and configuration, including manual adjustment of the redundant axis when desired. Conversely, during *observing* or *cutting* phases, the RL module is enabled and autonomously generates incremental actions on the SEW redundant degree of freedom to achieve workspace-aware posture reconfiguration while preserving the laparoscope pose. If a *blocked* state is detected, for instance, when two individuals are detected on opposite sides of the robot or when reliable perception of the workspace is not available (e.g., due to occlusions or degraded visual conditions), a motion inhibition mechanism is activated that suppresses autonomous commands and keeps the robot in its current configuration until a safe interaction state is restored.

Finally, at the execution level, the low-level controller converts the commanded redundant-axis updates into joint-space motion while enforcing kinematic feasibility and maintaining the laparoscope pose. Robot proprioceptive feedback, together with environment observations, is returned to the RL module to close the control loop.

Overall, the proposed system is organized as a hierarchical perception–decision–control architecture, where each layer operates at a distinct level of abstraction and fulfills a specific role in the interaction loop. The perception layer extracts contextual information from the environment, the decision-making layer determines the appropriate control mode based on the inferred human activity, and the control layer executes either manual or learning-based actions accordingly. The following sections describe these modules in detail, focusing on the human activity recognition system (Section 3.2) and the RL-based redundant-axis controller (Section 3.3).
**Algorithm 1** Hierarchical control loop for context-aware RL-based SEW reconfiguration  1:Initialize perception, decision-making, and control modules  2:**while** system is active **do**  3:    **// Perception and feedback**  4:    Acquire RGB-D data from Intel RealSense D455i  5:    Acquire robot pose and perception-derived environment feedback  6:    **// Decision-making layer**  7:    **if** occlusion is detected **then**  8:        mode ← *blocked*  9:    **else if** two individuals are detected on opposite sides of the robot **then**10:        mode ← *blocked*11:    **else if** gamepad_on **then**12:        mode ← *controlling*13:    **else if** scalpel_on **then**14:        mode ← *cutting*15:    **else**16:        mode ← *observing*17:    **end if**18:    **// Control action selection**19:    **if** mode = *controlling* **then**20:        Enable gamepad-based robot control21:        Disable RL-based SEW reconfiguration22:        $u_{t}\leftarrow$ gamepad command (control input to the robot)23:    **else if** mode = *blocked* **then**24:        Disable RL-based SEW reconfiguration25:        Activate motion inhibition26:        $u_{t}\leftarrow$ zero/stop command27:    **else**28:        Enable RL-based SEW reconfiguration29:        Construct state $s_{t}$ from robot pose and perception-derived environment feedback30:        Compute action $a_{t}=\pi \left(s_{t}\right)$31:        $u_{t}\leftarrow$ RL-based SEW update (pose-preserving motion)32:    **end if**33:    **// Execution**34:    Send robot motion commands $u_{t}$35:**end while**

### 3.2. Human Activity Recognition

Human activity recognition is used to provide contextual information that regulates the level of autonomy granted to the robot. Rather than attempting to recognize fine-grained surgical gestures, the proposed approach focuses on identifying a reduced set of high-level activity modes that are relevant for workspace adaptation and safety.

Visual perception is implemented using a modular computer vision pipeline based on the YOLO (You Only Look Once) architecture [47], selected for its ability to perform real-time object detection with low computational overhead. Instead of training a single multi-class model, the perception system employs separate models for detecting people, the robot control gamepad, and a scalpel. This design choice reflects a trade-off between computational efficiency and system flexibility; while a unified multi-task model could reduce inference redundancy, it would require joint training on heterogeneous datasets with potentially unbalanced class distributions, increasing model complexity and reducing robustness to domain shifts. In contrast, the use of specialized detectors enables independent optimization of each task, facilitates incremental system updates, and improves fault isolation. From a scalability perspective, new objects or interaction cues can be incorporated by adding dedicated models without retraining the entire perception system. This modular design simplifies training and validation while supporting independent maintenance, incremental integration of new components, and robust system-level verification.

Surgeon detection and tracking are performed using a pretrained YOLO-based pose estimation model that identifies the *person* class and provides bounding boxes for each detected individual in the scene. Since the baseline model provided satisfactory performance in the experimental environment, no additional fine-tuning was required. To reduce false positives, a minimum confidence threshold of 0.70 was applied, and detections below this value were discarded.

For each detected person, the geometric center of the bounding box is computed from the coordinates $\left(x_{1},y_{1},x_{2},y_{2}\right)$ as in Equation (1).

(1) $$c_{x}=\frac{x_{1}+x_{2}}{2},\;c_{y}=\frac{y_{1}+y_{2}}{2}$$

The horizontal coordinate $c_{x}$ is used to determine the relative lateral position of the individual in the scene. The image plane is divided into two vertical regions, left and right, based on the horizontal coordinate. The division is defined with respect to the image width *W*, such that $c_{x}<0.5\cdot W$ corresponds to the left region and $c_{x}\geq 0.5\cdot W$ to the right region, with the robot positioned near the image center. This classification allows the system to determine the side of the workspace occupied by the operator.

**Figure 4:**
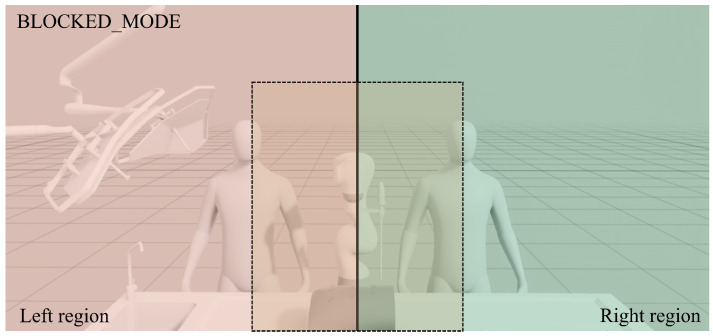
*Blocked* mode activation based on spatial human distribution. The image plane is partitioned into left and right regions according to the horizontal coordinate $c_{x}$ relative to the image width *W*. A central rectangular area (dashed area), defined by *c_left*, *c_right*, *c_top*, and *c_bottom*, represents the critical HRI zone around the robot workspace. When individuals are simultaneously detected on both sides of the robot within this region, the situation is classified as unsafe, triggering the *blocked* mode and inhibiting autonomous SEW axis adaptation.

In addition to this lateral partitioning, a rectangular central region is defined to represent the potential interference zone between the human workspace and the robot operating area. The boundaries of this region are defined by four parameters (*c_left*, *c_right*, *c_top*, *c_bottom*). This region corresponds to approximately 30% of the image width and 80% of the image height, centered around the robot workspace. When one or more individuals are detected inside this region, their lateral positions are evaluated. If people are detected simultaneously on opposite sides of the robot within this critical area, the system interprets the situation as potentially unsafe and activates a *blocked* mode that disables autonomous robot adaptation (see Figure 4). This region operates at the supervisory decision level, determining when the system transitions to a safety state rather than directly influencing motion generation. As such, its size modulates the conservativeness of the safety mechanism; enlarging the region leads to earlier activation of the *blocked* mode, while reducing it allows a wider operational workspace. Therefore, its impact is primarily on the operational envelope of the system rather than on the underlying control behavior.

The *blocked* state is also used to handle perception uncertainty arising from sensor limitations. In particular, situations in which no human is reliably detected within the workspace, potentially due to occlusions, partial visibility, or illumination variations, are conservatively interpreted as unsafe.

Rather than attempting to explicitly classify the cause of the perception failure, the system adopts a safety-first strategy: the absence of expected detections in the interaction region triggers the *blocked* mode, which inhibits autonomous robot motion until reliable perception is restored. This mechanism provides an implicit error-handling strategy that ensures safe behavior under degraded sensing conditions without requiring explicit occlusion modeling.

In parallel, two additional YOLO-based detectors are trained to recognize task-relevant objects. A dedicated model is trained to detect the robot control console using two labels: *console_on*, corresponding to situations in which the operator holds the console, and *console_off*, representing the console resting on a surface. The training dataset consists of 300 annotated images per class captured using the Intel RealSense D455i (Intel Corporation; Santa Clara, CA, USA) camera. The dataset is split into 80% training and 20% validation subsets, and the model is trained for 100 epochs with an input resolution of 640×640 pixels.

A second detector is trained to recognize the surgical instrument used during the experiments. In this case, only one class (*scalpel_on*) is defined, corresponding to situations in which the surgeon actively holds the instrument. The complementary class (*scalpel_off*) is intentionally omitted because the white color of the instrument makes reliable detection difficult when it is placed on surfaces with a similar appearance, which significantly increases the likelihood of false positives.

**Figure 5:**
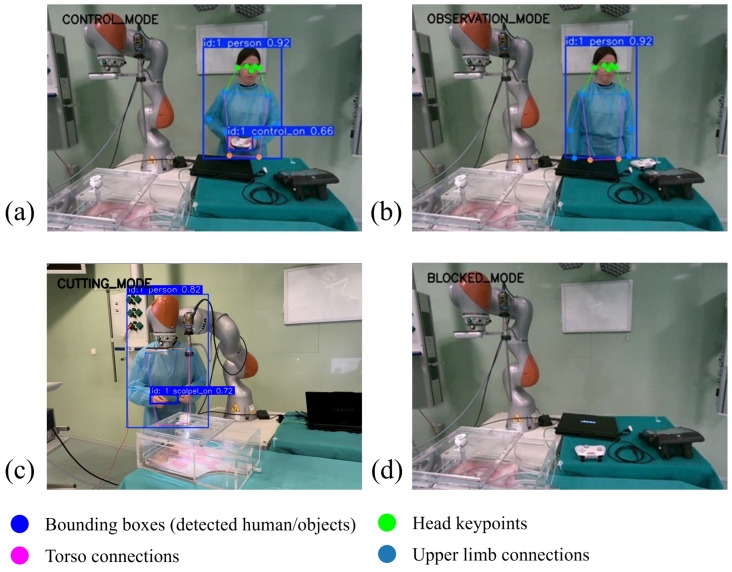
Human activity recognition outputs for different operational modes. (**a**) *Control mode*: the operator actively controls the manipulator using the gamepad. (**b**) *Observation mode*: the operator is present but not directly controlling the robot. (**c**) *Cutting mode*: the operator holds an scalpel within the workspace. (**d**) *Blocked mode*: two individuals are detected simultaneously on opposite sides of the robot within the interaction area, triggering the safety mechanism that inhibits autonomous robot adaptation.

The outputs of these perception modules are combined through a deterministic decision logic that assigns the interaction context to one of four activity modes: *controlling*, *observing*, *cutting*, or *blocked*. The *blocked* mode is evaluated first in the decision logic and is activated whenever a potentially unsafe or unreliable perception condition is detected, such as when two individuals are located on opposite sides of the robot within the interaction area, or when reliable perception of the workspace is not available (e.g., due to occlusions, or an absence of human detections). If no *blocked* condition is triggered, the *controlling* mode is activated when the gamepad is detected in the operator’s hand, indicating manual robot operation. The *cutting* mode is triggered when the system detects that a surgeon or an assistant surgeon is holding the scalpel next to the robotic platform. Cutting has been used as an example for this study, although the system can be implemented for any other surgical assistance task, such as trocar insertion or the use of laparoscopic assistance instruments. When neither of these conditions holds, and the surgeon remains present in the workspace, the system assigns the *observing* mode. This mode is activated whenever the spatial configuration of detected individuals indicates a potentially unsafe situation. The outputs of the four perception modules are illustrated in Figure 5.

The resulting models achieved high detection reliability in the experimental setup, with precision and recall values above 0.9 for the scalpel detector and high precision (above 0.95) with moderate recall for the gamepad detector. The overall detection performance, measured in terms of mAP@0.5, exceeded 0.85 for both tasks, ensuring robust identification of interaction cues required for mode selection. The perception pipeline operated at 8–9 Hz, with an average inference latency of about 110 ms. While this latency introduces a delay in the perception of interaction cues, its impact on the overall control behavior is mitigated by the hierarchical decision logic and the nature of the downstream control actions, as discussed in Section 3.3.2, where control operates on lower-bandwidth posture adaption rather than high-frequency motion commands. These results were considered sufficient for the purposes of real-time activity recognition within the proposed control framework. Despite occasional missed detections, the downstream decision logic incorporated temporal consistency and logical filtering, which mitigated the impact of isolated perception errors on the overall system behavior.

These activity states regulate the level of autonomy granted to the robot. The *controlling* and *blocked* modes immediately disable RL-based adaptation, ensuring full human authority or safety interruption. Conversely, the RL controller is allowed to operate only during *observing* and *cutting* phases, where autonomous redundancy adaptation can increase workspace clearance without interfering with active robot manipulation.

### 3.3. Reinforcement Learning Control

#### 3.3.1. Markov Decision Process

RL [16] is a type of machine learning in which an agent learns to interact with its environment to maximize the rewards it receives in the long term. This interaction learning problem is usually modeled as a Markov Decision Process (MDP).

In an MDP, sequential decision-making is modeled as a stochastic, agent-driven interaction with the environment. Each action selected and executed by the agent not only affects the immediate reward but also shapes the subsequent state of the system and, consequently, future rewards. Conceptually, an MDP can be described in terms of three core components: the state in which the agent is, the action it selects, and the agent’s ultimate goal. In their simplest forms, the elements are usually represented by the following tuple (2):

(2) $$[S,A,P\left(s_{t+1}|s_{t},a_{t}\right),R\left(s_{t},s_{t+1},a_{t}),\gamma \right]$$

where *S* denotes the set of possible states of the agent and *A* is the set of actions. $P(s_{t+1}|s_{t},a_{t})$ is the probability of transition to a future state $s_{t+1}$, when the agent is in state $s_{t}$ and applies action $a_{t}$. $R(s_{t},s_{t+1},a_{t})$ is the reward the agent expects to obtain when it transits from state $s_{t}$ to state $s_{t+1}$, and it is calculated using the reward function. Finally, γ is the discount factor of the reward function.

A policy $\pi (a_{t}|s_{t})$ is a mapping between states and probabilities of selecting each possible action. This process can be defined using sequence (3) as follows:

(3) $$s_{0},a_{0},r_{0},s_{1},a_{1},r_{1},s_{2},a_{2},r_{2},\ldots$$

#### 3.3.2. State Space

The state space *S* encodes the information required to describe the current configuration of the robot and its spatial relationship with the surgeon. This information is obtained from both the visual perception system and internal robot variables.

Under the Markov assumption, the current state is considered sufficient to describe the interaction context and therefore defines a fully observable MDP. In the proposed framework, this assumption applies to the operational modes in which the RL controller is active, namely the *observing* and *cutting* modes. The activation of the RL policy is handled by a high-level human activity recognition module, which disables the controller when reliable perception of the workspace is not available (e.g., due to occlusions, partial visibility, or absence of reliable human detections in the interaction region) by assigning the *blocked* mode. Consequently, the RL policy is only executed under conditions where the state can be considered reliably observable, while situations of partial observability are handled outside the MDP through this gating mechanism. The state vector at time step *t* is defined in Equation (4):

(4) $$s_{t}=\left[d_{t},side_{t},\epsilon_{t},{\dot{\epsilon }}_{t}\right]$$

The variable $d_{t}$ represents the average lateral distance between the surgeon and the robot. The surgeon’s position is estimated using the YOLO-based pose tracking system described in Section 3.2. From this model, the three-dimensional coordinates ($x_{c},y_{c},z_{c}$) of eight anatomical landmarks of the upper body are obtained, including wrists, elbows, shoulders, and hips, expressed in the camera reference frame. These coordinates are subsequently transformed into the robot reference frame ($x_{r},y_{r},z_{r}$).

Since SEW axis reconfiguration primarily affects the robot posture along the lateral direction, only the lateral coordinate $y_{r}$ of each transformed landmark is considered. The mean value of these coordinates is then computed to obtain a representative estimate of the lateral proximity between the surgeon and the robot. The distance *d* of each transformed landmark is considered. The mean value of these coordinates is then computed to obtain a representative estimate of the lateral proximity between the surgeon and the robot. The distance $d_{t}$ is defined as the absolute value of this mean lateral offset, ensuring that the magnitude of the separation is captured independently of the side from which the surgeon approaches. Since $d_{t}$ is derived from the perception pipeline described in Section 3.2, it is subject to the associated sensing latency. However, in the proposed framework, this delay does not critically affect control stability. The RL policy operates on the redundant SEW angle through incremental updates as defined in Section 3.3.3, rather than commanding high-frequency task-space motions. As a result, the control action corresponds to a low-bandwidth posture adaptation process. Moreover, as $d_{t}$ is computed as an average over multiple anatomical landmarks, it provides a smooth interaction signal and reduces sensitivity to short-term perception delays. Consequently, the system remains robust under the observed latency in the considered HRI scenario.

The variable $side_{t}$ is a discrete indicator that specifies the relative lateral side of the surgeon with respect to the robot. It takes values in the set {−1,+1}, where −1 denotes that the surgeon is located on the left side of the robot and +1 denotes that the surgeon is located on the right side. This variable allows the system to distinguish between spatial configurations that are equivalent in terms of distance but occur on opposite sides of the robot. Such information is essential for determining the correct direction of SEW axis reconfiguration.

The term $\epsilon_{t}$ denotes the current SEW angle, which characterizes the redundant configuration of the robotic arm. This variable directly represents the posture of the manipulator and therefore provides key information about the current redundancy state of the robot. In the implemented system, the SEW angle is constrained within the interval $\epsilon_{t}\in \left[-95^{{}^{\circ}},95^{{}^{\circ}}\right]$. This range was selected because it provides sufficient lateral reconfiguration to free workspace for the surgeon or nurse while avoiding operation near the extreme SEW limits, where the robot posture may become less suitable for stable and safe collaborative interaction.

Finally, ${\dot{\epsilon }}_{t}=\epsilon_{t}-\epsilon_{t-1}$ represents the temporal variation of the SEW angle between two consecutive time steps. This quantity provides information about the motion dynamics of the redundant axis and allows the agent to evaluate both the direction and smoothness of the executed actions. No temporal smoothing window is applied when computing this term, since $\epsilon_{t}$ is an internal control variable of the robot rather than a noisy perceptual signal. Consequently, the instantaneous difference between consecutive measurements provides a sufficiently stable estimate of the angular velocity of the SEW axis.

#### 3.3.3. Action Space

The action space *A* is defined as a discrete set composed of seven possible reconfigurations of the robot’s SEW axis, which correspond to decreasing, maintaining, or increasing the current SEW angle.

These actions are encoded as relative variations of the SEW angle, allowing the agent to perform progressive and controlled adjustments of the robot posture while avoiding abrupt motions. Formally, the action applied at time step *t* is defined in Equation (5):

(5) $$a_{t}=\Delta \epsilon_{t},$$

where $\Delta \epsilon_{t}$ represents a relative variation of the SEW angle. The action set consists of seven discrete increments defined in Equation (6):

(6) $$\Delta \epsilon_{t}\in \left\{-3^{{}^{\circ}},-2^{{}^{\circ}},-1^{{}^{\circ}},0^{{}^{\circ}},1^{{}^{\circ}},2^{{}^{\circ}},3^{{}^{\circ}}\right\}$$

This incremental formulation enables smooth posture adaptation by limiting the magnitude of each SEW update and avoiding abrupt robot motions.

#### 3.3.4. Reward

(a) Traditional dense reward

The traditional dense reward formulation is based on explicit criteria related to the spatial relationship between the surgeon and the robot, as well as the behavior of the SEW axis. This formulation directly evaluates whether the executed action moves the robot toward a desirable configuration and penalizes undesirable motion patterns. At each time step *t*, the reward is formalized by Equation (7):

(7) $$r_{t}=r_{track}+r_{smooth}+r_{dir,t},$$

where $r_{track}$ represents the main tracking term that guides the robot toward an appropriate SEW posture, $r_{smooth}$ penalizes abrupt variations of the SEW angle, and $r_{dir,t}$ evaluates whether the executed action modifies the SEW angle in the correct direction.

The $r_{track}$ term corresponds to the main component of the reward and aims to guide the robot toward an appropriate SEW posture depending on the proximity of the surgeon or nurse. This term is defined in Equation (8):

(8) $$r_{track}=-w_{e}\cdot {\left(\frac{err}{\epsilon_{max}}\right)}^{2}$$

where $w_{e}$ is a weighting factor that determines the importance of the tracking objective, $\epsilon_{max}$ represents the maximum allowable SEW inclination, and err denotes the angular error. In the implemented system, the parameters are set to $w_{e}=6.0$ and $\epsilon_{max}=\pm 95$, corresponding to the feasible redundancy range of the robot while maintaining the laparoscope pose. The angular error err is defined as the difference between the current SEW angle $\epsilon_{t}$ and a desired SEW angle $\epsilon^{*}(d_{t}$), as formalized in Equation (9):

(9) $$err=\epsilon_{t}-\epsilon^{*}\left(d_{t}\right)$$

The desired SEW angle $\epsilon^{*}(d_{t}$) is computed as a function of the lateral distance between the surgeon and the robot (see Equation (10)).

(10) $$\epsilon^{*}\left(d_{t}\right)=\left\{\begin{array}{cc} 0, & d_{t}\geq d_{safe}\\ side_{t}\cdot \epsilon_{max}\cdot {\left(\frac{d_{safe}-d_{t}}{d_{safe}-d_{max\_incl}}\right)}^{2}, & d_{max\_incl}<d_{t}<d_{safe}\\ side_{t}\cdot \epsilon_{max}, & d_{t}\leq d_{max\_incl}, \end{array}\right.$$

where $side_{t}$ indicates the relative side of the surgeon with respect to the robot, $d_{t}$ represents the lateral distance between them, $d_{safe}$ defines the distance above which no SEW inclination is required, and $d_{max\_incl}$ corresponds to the distance at which the maximum inclination should be reached.

In the proposed implementation, the parameters are set to $d_{safe}=0.8$ m and $d_{max\_incl}=0.45$ m, which corresponds to the saturation point to which the maximum SEW inclination is applied. Note that the distance $d_{t}$ represents the average lateral distance of multiple tracked upper-body keypoints rather than the minimum distance between the human and the robot. Consequently, when $d_{t}$ approaches 0.45 m, some anatomical landmarks may already be closer to the robot workspace. This formulation therefore provides a conservative estimate of human proximity and ensures early posture adaptation to preserve safe workspace clearance.

This formulation induces three distinct behaviors depending on the surgeon’s or nurse’s lateral distance. For distances greater than $d_{safe}$, the target SEW angle remains zero, maintaining the robot in a neutral posture. When $d_{max\_incl}<d_{t}<d_{safe}$, the desired angle increases progressively, producing a smooth posture adaptation as the surgeon approaches the robot. Finally, when the distance becomes smaller than $d_{max\_incl}$, the desired SEW angle saturates at its maximum value.

The second component $r_{smooth}$ evaluates the smoothness of the SEW axis motion. Since the action space is discretized into seven possible actions, it is necessary to regulate the magnitude of the executed updates in order to prevent abrupt posture changes. This term is defined in Equation (11):

(11) $$r_{smooth}=-w_{v}\cdot {\dot{\epsilon_{t}}}^{2},$$

where ${\dot{\epsilon }}_{t}$ represents the angular velocity applied to the SEW axis and $w_{v}$ is a weighting factor that determines the importance of motion smoothness within the reward function. In this study, this parameter is set to $w_{v}=0.2$. By penalizing large angular variations, this term encourages gradual posture adjustments and contributes to generating smooth and stable SEW reconfigurations during HRI.

Lastly, the directional term $r_{dir,t}$ evaluates whether the action executed by the agent modifies the SEW axis in the appropriate direction, that is, whether the applied change contributes to reducing the error between the current angle $\epsilon_{t}$ and the desired angle $\epsilon^{*}\left(d_{t}\right)$. Unlike the tracking term $r_{track}$, this component does not directly penalize the magnitude of the error but rather the coherence between the direction of motion and the corrective direction required to reduce it. This term is defined in Equation (12):

(12) $$r_{dir,t}=w_{d}\cdot tanh\left(\dot{\epsilon_{t}}\right)\cdot tanh\left(\frac{-err}{err_{scale}}\right),$$

where $err_{scale}$ acts as normalization factor that regulates the sensitivity of the directional term, preventing excessively large contributions caused by high angular variations or large tracking errors. In the proposed implementation, this parameter is set $err_{scale}=20.0$. The parameter $w_{d}=1.5$ determines the weight assigned to the directional component within the reward function.

The first factor encodes the direction of the motion executed by the robot, while the second factor reflects the direction of the error that must be corrected. The product of these two terms allows the reward to distinguish whether the executed action reduces or increases the angular deviation. When the sign of the motion is consistent with the correction required to reduce the error, the contribution becomes positive; otherwise, it becomes negative.

(b) Fuzzy logic-based reward

To introduce a greater degree of flexibility in the evaluation of the agent’s actions, a variant of the reward function based on fuzzy logic is developed. Fuzzy logic enables reasoning under uncertainty by mapping crisp input variables into degrees of membership over linguistic categories in the interval [0, 1] [48]. This representation allows the use of soft thresholds and smooth interpolation between overlapping regions of the state space. As a result, the proposed approach models the transitions between favorable and unfavorable states more gradually, reducing the sensitivity of the system to small variations in the state variables.

In this study, rather than defining a fully fuzzy reward function from scratch, fuzzy logic is used as a gain scheduling mechanism that modulates a weighting term. In particular, the modification is applied exclusively to the tracking term $r_{track}$, since it is the only component whose contribution depends directly on the spatial relationship between the surgeon and the robot. In this case, fuzzy logic allows the intensity of the penalty to be adapted continuously as a function of the human–robot distance. In contrast, the terms $r_{smooth}$ and $r_{dir,t}$ describe well-defined criteria through analytical expressions, and therefore a fuzzy formulation does not provide a clear advantage.

Consequently, the proposed formulation preserves the same structure of the tracking term $r_{track}$ but replaces the constant weight $w_{e}$ with a distance-dependent weight $w_{f}\left(d_{t}\right)$. The fuzzy tracking reward is therefore defined in Equation (13):

(13) $$r_{track}^{fuzzy}=-w_{f}\left(d_{t}\right)\cdot {\left(\frac{err}{\epsilon_{max}}\right)}^{2},$$

where $w_{f}\left(d_{t}\right)$ is obtained through a fuzzy inference system based on the lateral distance $d_{t}$ between the surgeon and the robot.

**Figure 6:**
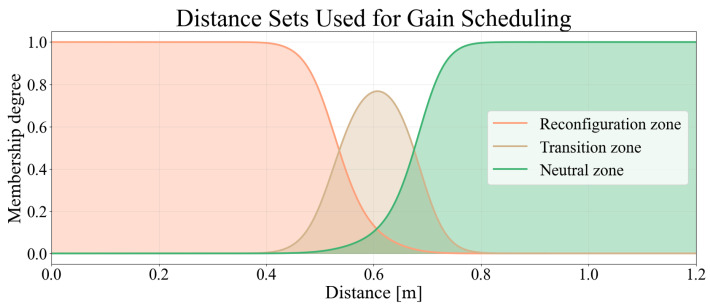
Fuzzy membership functions for gain scheduling based on distance.

The fuzzy system uses the lateral distance $d_{t}$ as input and defines three linguistic sets: *reconfiguration zone*, *transition zone*, and *neutral zone*, as illustrated in Figure 6. Each set describes a region of the workspace in which the robot should exhibit a different posture adaptation behavior. In particular, the *reconfiguration zone* corresponds to situations where the human is close to the robot and a high SEW reconfiguration is desirable, while the *neutral zone* corresponds to situations where no significant posture adjustment is required. The *transition zone* is intentionally defined with a maximum membership value lower than unity to avoid dominance over the extreme regions and to ensure a smooth interpolation between *reconfiguration* and *neutral* behaviors, preventing abrupt changes in the scheduled gain.

From the membership degrees associated with these sets, a distance-dependent weight $w_{f}\left(d_{t}\right)$ is computed. This value is obtained through a Sugeno-type fuzzy interpolation, as defined in Equation (14):

(14) $$w_{f}\left(d_{t}\right)=\mu_{reconf}\cdot w_{reconf}+\mu_{trans}\cdot w_{trans}+\mu_{neutral}\cdot w_{neutral},$$

where $\mu_{reconf}\left(d_{t}\right)$, $\mu_{trans}\left(d_{t}\right)$, and $\mu_{neutral}\left(d_{t}\right)$ denote the degree of membership of the distance $d_{t}$ to the fuzzy sets *reconfiguration zone*, *transition zone*, and *neutral zone*, respectively, and $w_{reconf}$, $w_{trans}$, and $w_{neutral}$ are the consequences of the corresponding rules. In the proposed implementation, these values are set such that $w_{f}\left(d_{t}\right)\in \left[4.8,6.5\right]$, where the lower bound corresponds to full activation of the *neutral zone*, the intermediate value $w_{f}=6.0$ is associated with the peak of the *transition zone*, and the upper bound corresponds to full activation of the *reconfiguration zone*.

With this strategy, when the surgeon or nurse is close to the robot (high membership to *reconfiguration zone*), the system increases the weight $w_{f}$, penalizing the angular error more strongly and encouraging a larger SEW axis correction. Conversely, when the surgeon is far from the robot (high membership to *neutral zone*), the weight decreases, reducing the penalty and preventing unnecessary posture adjustments. For intermediate distances (*transition zone*), the resulting weight produces a smooth transition between these two behaviors.

In this way, the fuzzy logic formulation does not introduce new evaluation criteria but modifies how the tracking penalty is quantified, while in the traditional dense reward, the tracking term penalizes the posture error through a fixed analytical expression, the fuzzy formulation dynamically adapts the intensity of the penalty as a function of the human–robot distance, providing a smoother and more context-aware response. All shared components of the reward function (e.g., effort, safety barrier, and symmetry terms) were kept identical across experiments, and no formulation-specific hyperparameter tuning was performed. The reported results therefore isolate the effect of the alignment-related term.

The reported parameters were selected for the present proof-of-concept and should therefore be interpreted as design parameters tied to the current robot platform, sensing setup, and operating-room mock-up.

## 4. Results

### 4.1. Training

Training was conducted directly in the real-world experimental setup to avoid the limitations associated with the simulation-to-reality gap [49]. The RL policy was trained using a Deep Q-Network (DQN) agent [50] implemented through the *skrl* library [51]. The selection of this RL agent was motivated by its suitability for discrete action spaces and its widespread adoption in RL-based motion planning [15], where it has demonstrated stable convergence and reliable performance, making it an appropriate baseline for evaluating the proposed reward formulations. By restricting the action space to predefined increments, the control policy inherently limits abrupt or unstable behavior, which is desirable in close HRI. In addition, DQN provides stable and sample-efficient learning in discrete domains, making it a practical choice for real-world training, where data collection is time-consuming and subject to physical constraints. Communication with the robotic manipulator was achieved by extending the open-source *libiiwa* interface [52], which was used to send motion commands and retrieve robot state information.

To improve the robustness and generalization capability of the learned policy, domain randomization was applied during training. At each environment reset, the Cartesian position of the robot was randomized within predefined ranges: x∈[500, 580] mm, y∈[−40, 40] mm, and z∈[600, 655] mm. This strategy exposes the agent to slightly different initial configurations and is commonly used to prevent overfitting to a single workspace configuration [53].

In addition, to avoid requiring a human-in-the-loop during the training process, a dataset of prerecorded human motions was used to emulate the presence of a surgeon or a nurse. Specifically, ten video sequences of two minutes each were recorded using the RGB-D perception system. These videos capture realistic upper-body movements of a person approaching and moving away from the robot under different interaction scenarios. During training, one of these recordings was randomly selected and replayed, allowing the perception module to generate the corresponding distance used by the RL agent.

All RL training and evaluation experiments were executed on a mini desktop computer equipped with an AMD Ryzen 5 7430U processor (up to 4.3 GHz), 32 GB of RAM, and a 1 TB NVMe SSD, running Ubuntu Linux. The processor features 8 cores and 16 threads, which were used to execute the perception, control, and RL modules concurrently.

Additional details regarding the hyperparameters selected for the agent are presented in Table 1. The DQN policy was implemented using a fully connected neural network composed of two hidden layers with 128 and 64 neurons, respectively.

**Figure 7:**
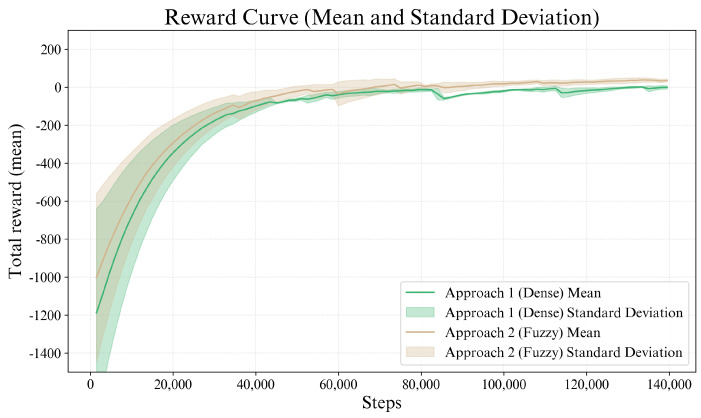
Mean and standard deviation of the cumulative training rewards obtained over ten runs for the dense and fuzzy reward formulations. The solid lines represent the mean values, while the shaded regions correspond to one standard deviation computed across runs.

Figure 7 shows the mean and standard deviation of the rewards obtained for both reward formulations (dense and fuzzy), computed over ten training sessions for each approach. Each training session consisted of 140,000 interaction time steps and was initialized with a different random seed to ensure variability across sessions. The fuzzy reward formulation demonstrates slightly faster early learning compared to the traditional dense reward, as indicated by the higher reward values observed during the first 20,000–30,000 steps. This behavior suggests that the adaptive weighting mechanism introduced by the fuzzy system provides a smoother optimization landscape during the initial exploration phase.

The quantitative metrics reported in Table 2 further support these observations. Both approaches achieve a perfect success rate of 1.00, indicating that the agents reliably learn the desired SEW reconfiguration behavior. However, the fuzzy reward formulation exhibits improved training stability. In particular, the tail standard deviation is reduced from 14.03 in the dense formulation to 10.62 in the fuzzy formulation, indicating a reduction of over 24% in variability during the final stages of training. In this study, the tail region is defined as the final 35% of the training steps. This choice is motivated by the analysis of the learning curves, which shows that both approaches reach an approximately stationary regime after about 60–70% of the total training process. Therefore, considering the last 35% makes it possible to characterize this stationary regime consistently, while avoiding transient phases of learning and reducing the influence of local fluctuations in only the very last portion of training.

A similar trend is reflected in the tail variation coefficient, which decreases from 1.1 to 0.53, suggesting that the fuzzy approach yields a reduction of over 50% in relative variability once convergence is reached.

The stability ratio also slightly improves for the fuzzy reward compared to the dense formulation, indicating reduced oscillations in the learned behavior. In terms of convergence speed, both approaches exhibit comparable performance. The dense reward reaches the 80% performance threshold slightly earlier (31,000 steps) than the fuzzy formulation (33,500 steps), whereas the fuzzy reward achieves the 90% threshold faster (45,000 steps compared to 50,500 steps).

Finally, the normalized area under the learning curve (AUC) is nearly identical for both approaches ($8.28\times 10^{-1}$ for the dense reward and $8.25\times 10^{-1}$ for the fuzzy reward), suggesting that the overall training efficiency of the two formulations is comparable. Nevertheless, the lower variability and improved stability metrics observed for the fuzzy formulation indicate that it provides a more consistent learning process while maintaining equivalent final performance.

### 4.2. Evaluations

**Figure 8:**
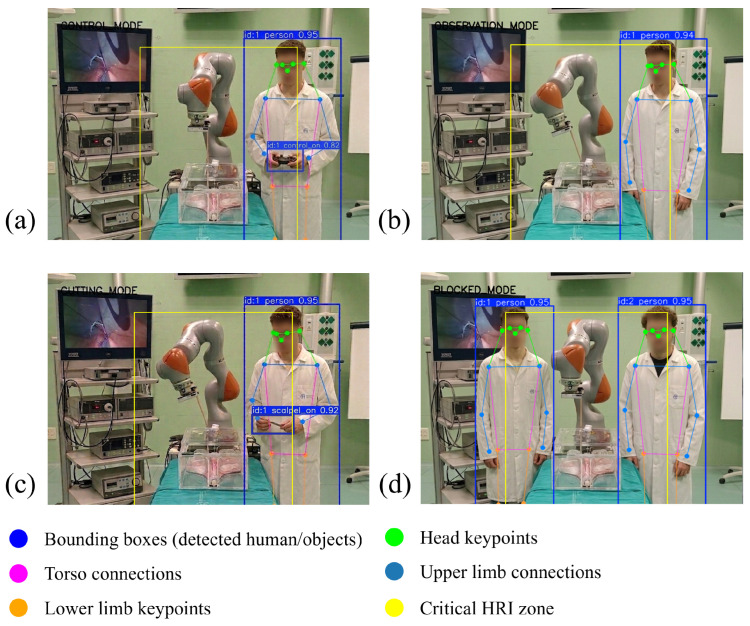
Real-world evaluation trials of the proposed framework under different interaction conditions. The perception outputs are overlaid on the RGB image, including blue bounding boxes for detected individuals and objects with associated confidence scores, yellow bounding boxes for interaction region, and skeletal keypoints for human pose estimation. (**a**) *Control mode*: manual operation is detected and autonomous adaptation is disabled. (**b**) *Observation mode*: no explicit interaction cues are detected, the robot autonomously reconfigures its posture to increase workspace around the surgeon. (**c**) *Cutting mode*: the system maintains adaptive behavior while the surgeon manipulates a surgical tool. (**d**) *Blocked mode*: the presence of two humans within the interaction area triggers a safety condition that inhibits robot motion.

Consistent with the results obtained in Section 4.1, the best weights from the best learning curves of each approach were used in the evaluation trials. During each evaluation trial, the robot interacted with a real human whose upper-limb body was continuously monitored through the vision-based tracking system described in Section 3.2. During these interactions, the distance between the human and the robot varied randomly over time as the human approached or moved away from the manipulator. For safety, external torque monitoring was enforced on joints 2–5. In the present implementation, conservative initial thresholds of 18, 12, 12, and 8 N·m were defined for joints 2 to 5, respectively. If any of these thresholds were exceeded, the robot triggered a safety stop. These values were selected to remain above the torque sensing uncertainty while ensuring sufficient sensitivity to unexpected contacts during low-speed collaborative operation. Final threshold tuning should nevertheless be validated experimentally with respect to the expected transient and quasi-static interaction conditions of the application. Figure 8 shows representative experimental results. As supplementary material, a video of the real-world evaluation trials is available at https://youtu.be/eAhHK8B4io8 (accessed on 28 April 2026).

To assess the effectiveness of the learned behavior, two complementary evaluation metrics were defined: directional response accuracy and task compliance rate. These metrics evaluate both the qualitative correctness of the robot’s reflex response and the quantitative alignment of the robot configuration with the desired task behavior. For each metric and each approach, ten evaluation runs were performed, resulting in a total of twenty evaluations. Each evaluation consisted of 500 time steps and was initialized with a different random seed to ensure variability across trials and avoid bias in the reported performance metrics. To ensure balanced coverage of the workspace, five runs were conducted with the human located on the right side of the robot and five on the left side. During all evaluation trials, no collisions between the robot and the human operator were observed, including scenarios involving close-proximity interaction.

#### 4.2.1. Directional Response Accuracy

Directional response accuracy evaluates whether the robot reacts in the appropriate qualitative direction in response to the human motion. In the considered interaction scenario, the expected reflex behavior of the robot is straightforward: when the human approaches the robot, the manipulator should progressively tilt away from the human in order to free workspace, whereas when the human moves away, the robot should gradually recover its neutral configuration. If the human position remains approximately constant, the robot should avoid unnecessary reflex motion.

To determine the human motion tendency, the variation in the human–robot distance over a short temporal window is analyzed. The distance variation over *k* time steps is defined in Equation (15) as follows:

(15) $$\Delta d_{t}=d_{t}-d_{t-k},$$

where *k* represents a temporal smoothing window used to reduce the influence of measurement noise and small oscillations in the distance signal. In this work, k=5 time steps were used. Based on this variation, the human motion tendency is classified into three possible cases following Equation (16):

(16) $$tendency\left(t\right)=\left\{\begin{array}{cc} approaching, & \Delta d_{t}<-\tau_{d}\\ retreating, & \Delta d_{t}>\tau_{d}\\ static, & \mathrm{otherwise} \end{array}\right.$$

where $\tau_{d}$ denotes a distance deadband used to filter small variations that are not meaningful for the interaction. In these evaluations, a threshold of $\tau_{d}=0.02$ m was used.

Similarly, the variation of the SEW redundancy angle over the same temporal window is computed in Equation (17):

(17) $$\Delta \epsilon_{t}=|\epsilon_{t}\left|-\right|\epsilon_{t-k}|,$$

Because small fluctuations may appear in both the distance measurements and the robot configuration, not every time step provides meaningful information about the robot response. Therefore, the notion of eligible steps is introduced. A time step is considered eligible if the interaction state provides enough information to evaluate whether the robot responded correctly. Formally, a step *t* is considered eligible when either a meaningful change in human distance is observed, or when the robot exhibits a noticeable SEW motion even though the human remains approximately static (see Equation (18)):

(18) $$E_{t}^{dir}=\left\{\begin{array}{cc} 1, & \left|\Delta d_{t}\right|>\tau_{d}\\ 1, & \left|\Delta d_{t}\right|\leq \tau_{d}\wedge \left|\Delta \epsilon_{t}\right|>\tau_{\epsilon }\\ 0, & \mathrm{otherwise} \end{array}\right.$$

where $\tau_{\epsilon }$ denotes a deadband used to filter negligible SEW variations. A threshold of $\tau_{\epsilon }=1^{{}^{\circ}}$ was used in the evaluation.

For each eligible step, the robot response is considered correct if the SEW motion is consistent with the expected reflex behavior. In particular, when the human approaches the robot ($\Delta d_{t}<-\tau_{d}$), the magnitude of the SEW angle should increase in the evasive direction, indicating that the robot tilts away from the human. Conversely, when the human moves away ($\Delta d_{t}>\tau_{d}$), the robot should progressively return toward the neutral configuration, which corresponds to a decrease in the magnitude of the SEW angle. Finally, if the human position remains approximately unchanged ($\left|\Delta d_{t}\right|\leq \tau_{\epsilon }$), the robot should avoid unnecessary motion, implying that the SEW variation should remain within the predefined deadband.

Based on these conditions, directional response accuracy is computed as the proportion of eligible time steps in which the robot exhibits the correct qualitative response, formalized in Equation (19):

(19) $$\mathrm{Directional}\;\mathrm{Response}\;\mathrm{Accuracy}=\frac{\sum_{t=1}^{T}C_{t}^{dir}}{\sum_{t=1}^{T}E_{t}^{dir}}\times 100$$

where $E_{t}^{dir}$ indicates whether the step is eligible and $C_{t}^{dir}$ is a binary variable that takes the value 1 when the robot response is correct and 0 otherwise.

This metric therefore measures how consistently the controller reacts in the appropriate direction during the HRI, independently of the exact magnitude of the SEW angle.

Based on the metric definition, Table 3 summarizes the directional response accuracy obtained for both control approaches during the evaluation trials.

Approach 1 achieved a directional response accuracy of 90.24±3.26%, whereas Approach 2 reached 90.97±2.06%. These results suggest that Approach 2 provides a slightly more consistent qualitative reflex response to variations in the human–robot distance, yielding a higher proportion of correct directional reactions throughout the interaction.

#### 4.2.2. Task Compliance Rate

While directional response accuracy evaluates whether the robot reacts in the correct qualitative direction, it does not guarantee that the robot configuration remains consistent with the desired reflex posture throughout the interaction. For this reason, a second metric called task compliance rate is introduced. This metric evaluates how closely the robot maintains the expected SEW configuration for a given human–robot distance.

In the considered task, the desired robot behavior can be described through the reference SEW posture $\epsilon^{*}\left(d_{t}\right)$ previously defined in Section 3.3.4. When the human remains far from the robot, the manipulator should stay close to the neutral configuration. As the human approaches, the SEW angle should progressively increase in magnitude in order to free workspace on the human side. Conversely, when the human moves away again, the robot should gradually return toward the neutral configuration.

Because transient effects may appear at the beginning of each evaluation episode, a short initialization phase is excluded from the analysis. A step *t* is therefore considered eligible for the task compliance metric only after the warm-up period has elapsed. The eligibility condition is defined in Equation (20):

(20) $$E_{t}^{task}=\left\{\begin{array}{cc} 1, & t>t_{warmup}\\ 0, & \mathrm{otherwise} \end{array}\right.$$

where $t_{warmup}$ denotes the number of initial time steps excluded from the evaluation. In this work, the first $t_{warmup}=10$ steps were discarded.

For each eligible step, the robot configuration is considered compliant if the SEW angle remains sufficiently close to the desired reference posture defined in Equation (21). This condition is evaluated using a tolerance threshold that allows small deviations around the reference configuration:

(21) $$\left|\epsilon_{t}-\epsilon^{*}\left(d_{t}\right)\right|\leq \delta_{\epsilon },$$

where $\delta_{\epsilon }$ represents the maximum admissible deviation from the desired SEW posture. In the experiments, a tolerance of $\delta_{\epsilon }=5^{{}^{\circ}}$ was used.

Based on these conditions, the task compliance rate is defined as the proportion of eligible time steps in which the robot configuration satisfies the reference posture constraint, as formalized in Equation (22):

(22) $$\mathrm{Task}\;\mathrm{Compliance}\;\mathrm{Rate}=\frac{\sum_{t=1}^{T}C_{t}^{task}}{\sum_{t=1}^{T}E_{t}^{task}}\times 100$$

where $E_{t}^{task}$ indicates whether the step is eligible according to Equation (20), and $C_{t}^{task}$ is a binary variable that takes value 1 when the condition defined in Equation (21) is satisfied and 0 otherwise.

This metric therefore measures how consistently the robot maintains the desired SEW posture throughout the HRI, independently of the instantaneous direction of the motion.

Based on the metric definition, Table 4 summarizes the task compliance rate obtained for both control approaches during the evaluation trials.

Approach 1 achieved a task compliance rate of achieved a task compliance rate of 73.08±10.29%, whereas Approach 2 reached 77.78±6.52%. These results suggest that Approach 2 maintains a SEW configuration that is more consistent with the desired reflex posture throughout the interaction.

## 5. Discussion

Seven-degree-of-freedom manipulators inherently provide kinematic redundancy, enabling a continuum of arm configurations for a fixed end-effector pose. This redundancy is traditionally exploited to avoid singularities [54,55], motion planning [56,57], or stabilize impedance parameters [58,59].

In this work, a context-aware and proactive redundancy resolution proof-of-concept framework is introduced, transforming a collaborative laparoscope holder into an intent-driven spatial assistant. By integrating a modular vision-based human activity recognition pipeline with a gated RL controller, the system adapts the redundant SEW angle according to the operative context (*controlling*, *observing*, or *cutting*), enabling dynamic workspace reconfiguration without altering the laparoscope pose. This design departs from classical formulations by explicitly incorporating high-level contextual information into the redundancy resolution process, allowing the robot to anticipate human motion and reorganize the workspace prior to physical interaction.

From a conceptual perspective, the proposed framework shifts redundancy resolution from an analytical or reactive formulation toward a perception-conditioned and context-driven process. In contrast to classical null-space or hierarchical control strategies, where redundancy is resolved through predefined objectives or constraint hierarchies, the robot configuration is continuously modulated based on high-level semantic information about human activity and spatial proximity. This results in a form of context-dependent redundancy adaptation in which the internal kinematic configuration evolves in response to the perceived interaction state, rather than being solely determined by geometric or force-based criteria.

Moreover, the use of a model-free RL policy enables this adaptation without requiring explicit modeling of human motion or interaction dynamics, which are inherently uncertain and difficult to formalize in surgical environments. As a result, the system can adapt to the unpredictable and uncertain human behavior, while maintaining task constraints such as laparoscope pose preservation. This integration of perception, decision-making, and learning supports a more anticipatory form of behavior, where workspace reconfiguration is driven by inferred human intent rather than reactive responses to physical interaction.

From a learning perspective, the proposed fuzzy logic-based reward formulation achieves more stable and interpretable behavior, while the traditional dense reward provides a direct encoding of tracking, smoothness, and directional consistency, the fuzzy logic-based formulation introduces a distance-dependent gain scheduling mechanism that modulates the tracking penalty continuously. This formulation leverages the ability of fuzzy logic to handle uncertainty and imprecision in HRI scenarios [60]. In the context of robot-assisted surgery, this uncertainty is associated with the inherently variable and partially unpredictable motion of the surgeon and assisting staff, as well as with perception limitations such as occlusions, and partial visibility [42]. By mapping crisp perceptual inputs into degrees of membership over linguistic interaction zones, the reward function implements soft thresholds through overlapping membership functions, enabling smooth and continuous transitions between behavioral regimes and avoiding abrupt changes in the reward landscape typical of analytical formulations [40,61].

As shown in Section 4, this leads to improved training stability and reduced variability in the learned policy, suggesting that fuzzy reward shaping is particularly effective in handling the stochastic and non-stationary nature of human motion. Furthermore, the rule-based structure of the fuzzy formulation provides an implicit form of context-dependent gain scheduling, where the contribution of the tracking term is adaptively modulated according to the human–robot distance, resulting in a more robust response to perception noise and variability [61]. Overall, these results provide empirical support for the working hypothesis of this study. Agents trained with fuzzy logic-based rewards tended to outperform those trained with conventional dense reward formulations in terms of convergence speed and asymptotic stability. This behavior is consistent with prior findings in fuzzy RL, which highlight the advantages of smooth reward structures for improving adaptability and stability in dynamic and uncertain environments [18]. In addition, the linguistic and rule-based nature of the formulation provides a more transparent and interpretable structure compared to purely analytical reward definitions [18,60], which is particularly desirable in safety-critical HRI surgical contexts. Although this formulation does not redefine the task objective, it significantly improves convergence properties and robustness in HRI scenarios. Nonetheless, while the observed improvements are consistent with the proposed interpretation of the fuzzy reward as a distance-dependent and smooth shaping mechanism, a more detailed decomposition of its individual components (e.g., membership function design, overlap structure, and gain modulation) would provide further insight into their specific contributions to the overall performance.

From an application standpoint, the results indicate that the proposed approach can contribute to improved workspace allocation in the operating room, potentially reducing ergonomic strain and facilitating more natural interaction patterns. Unlike classical reactive compliance schemes, the presented architecture enables workspace reconfiguration prior to contact, while preserving the laparoscope pose and maintaining safety through a multi-layered mechanism combining perception-based motion inhibition and collision-triggered stops.

When compared with classical null-space-based methods, such as those employing safety-constrained compliance [40] or hierarchical operational space control [41], several distinctions emerge. These approaches provide strong analytical guarantees for task decoupling and force minimization, particularly at the RCM level. In particular, hierarchical formulations achieve strict separation between primary surgical tasks and secondary objectives, ensuring high precision and predictable behavior under interaction disturbances. However, these approaches fundamentally rely on reactive paradigms, where robot adaptation is triggered by physical forces or predefined constraints.

In contrast, the proposed framework introduces a perception-driven paradigm in which redundancy resolution is conditioned on inferred human activity and spatial context. This shift enables proactive workspace adaptation, allowing the robot to respond to surgeon or nurse intent rather than to physical perturbations, while this capability represents a qualitative advancement in HRI, it comes at the cost of reduced analytical guarantees compared to model-based approaches. In particular, the learned policy lacks the formal stability and constraint enforcement properties of hierarchical control, and its performance is inherently dependent on the reliability of the perception system. Consequently, a systematic quantitative benchmarking against classical redundancy resolution methods (e.g., null-space optimization and hierarchical control) is required to evaluate the trade-offs between analytical guarantees and perception-driven adaptive behavior under comparable experimental conditions.

A further limitation of the proposed framework is its dependence on several manually specified parameters, including perception thresholds, safety margins, reward weights, and fuzzy membership definitions. In the present proof-of-concept, these parameters were selected empirically to ensure stable and safe operation on the considered robot platform and laboratory operating-room setup. Although such calibration is common in real-world robotic systems, it may limit transferability across users, sensing configurations, or workspace geometries. Moreover, significant changes in these design choices may require partial or complete retraining of the RL policy, thereby reducing deployment flexibility. This limitation is not unique to the proposed approach, as classical redundancy-resolution and APF-based methods also rely on carefully tuned gains and objective weights; however, it remains an important practical challenge for learning-based systems.

Consequently, several avenues for future research are identified. First, future work should address the residual oscillatory behavior occasionally observed around nominal static configurations. Although limited in magnitude, these micro-adjustments may reduce motion naturalness and user confidence, especially in close HRI. A likely source of this effect is the sensitivity of the perception-to-policy pipeline to small fluctuations in visually estimated human position. This suggests the need for additional robustness mechanisms, such as temporal filtering, hysteresis in state transitions, improvements in the perception layer through multi-camera layouts, or hybrid control strategies that combine learning-based adaptation with additional consistency constraints. In this context, shielded RL [62] emerges as a promising direction, as it enables the incorporation of safety or behavioral constraints that can suppress undesirable actions, such as small oscillatory commands, while preserving the adaptive capabilities of the learned policy. Such developments should also be accompanied by user-centered assessments of perceived safety, trust, and workflow acceptability in order to verify the practical suitability of the proposed method.

Second, future work should investigate strategies to reduce parameter sensitivity and improve deployment robustness. Potential directions include adaptive or self-tuning reward formulations, broader domain randomization over task and environment parameters, preference-conditioned policies that allow high-level behavior modulation without retraining, and hierarchical or supervisory mechanisms capable of adjusting control objectives online.

Third, the present study focuses on the feasibility of context-aware redundancy adaptation as a control and HRI strategy, but it does not yet quantify its ergonomic benefit for clinical users. Since the ultimate motivation of the proposed framework is to improve workspace allocation and reduce the strain associated with awkward postures in the operating room, future research should include a dedicated ergonomic assessment. In particular, standardized observational methods such as the Rapid Upper Limb Assessment (RULA) [63] could be used to evaluate whether the proposed workspace-aware reconfiguration effectively reduces postural load for surgeons or assisting surgical staff during representative laparoscopic tasks.

Fourth, although DQN already provides a fine-grained control of the redundant degree of freedom, future work should also investigate the impact of action-space design on control performance, while the present study adopts a discrete formulation to ensure bounded and predictable behavior during real-world operation, continuous action-space methods based on actor–critic algorithms (e.g., deep deterministic policy gradient (DDPG) [64], proximal policy optimization (PPO) [65], or soft actor–critic (SAC) [66]) could provide higher control resolution and flexibility. A systematic comparison between discrete and continuous formulations will be explored to assess potential trade-offs between stability, safety, and control expressiveness in surgical HRI scenarios.

Finally, beyond these specific directions, the results of this study suggest that integrating perception-driven context awareness with learning-based control constitutes a promising pathway toward more intuitive and adaptive surgical robotic assistants. Bridging the gap between reactive control strategies and proactive, human-centered interaction remains a key challenge in the field. In this regard, future work should aim to consolidate the proposed framework with stronger guarantees on safety, robustness, and generalization, enabling its deployment in more complex and clinically relevant operating room scenarios.

## 6. Conclusions

This paper presents a perception-aware RL framework for workspace-aware posture adaptation in collaborative surgical robotics. By exploiting the redundant degree of freedom of a seven-degree-of-freedom manipulator, the proposed approach enables adaptive reconfiguration of the robot posture in response to the spatial relationship with the human operator, while preserving the primary task execution.

Unlike prior approaches that rely predominantly on force-based compliance or constraint-driven formulations, the proposed method integrates vision-based human activity recognition with a learning-based control policy, allowing for proactive and context-aware adaptation. In addition, a fuzzy logic-based gain scheduling mechanism is introduced to modulate the tracking component of the reward function, enabling smoother transitions between behavioral regimes and reducing sensitivity to small variations in the human–robot distance.

The framework was validated in a real-world experimental setup, where the learned policies demonstrated consistent and stable behavior across multiple evaluation trials. The results show that the robot is capable of generating appropriate reflexive responses to human motion while maintaining task compliance, supporting the effectiveness of combining RL with fuzzy modulation for adaptive HRI.

Despite these promising results, the main limitation of the proposed framework is the residual oscillatory behavior occasionally observed around nominal static configurations. Although limited in magnitude, these micro-adjustments may reduce motion naturalness and compromise the perceived smoothness of the robot response during close HRI.

Future work will therefore focus on improving the stability and robustness of the reconfiguration behavior through additional mechanisms such as temporal filtering, hysteresis in state transitions, and shielded RL formulations. In parallel, future research will investigate strategies to reduce sensitivity to manually specified parameters and improve deployment flexibility, while also evaluating the ergonomic implications of the proposed approach through dedicated user studies.

## Figures and Tables

**Table 1:** DQN hyperparameters.

Parameters	Value
Memory size	15,625
Batch size	64
Discount factor γ	0.99
Soft update τ	0.005
Learning rate η	$10^{-3}$

**Table 2:** Quantitative comparison of the dense and fuzzy reward formulations in terms of stability, convergence speed, and cumulative learning performance during training.

Approach	Tail Standard Deviation	Normalized AUC	Tail Variation Coefficient	Stability Ratio	Steps-to-80	Steps-to-90	Success Rate
Approach 1 (dense)	14.03	$8.28\times 10^{-1}$	1.10	$2.26\times 10^{-2}$	31,000	50,500	1.00
Approach 2 (fuzzy)	10.62	$8.25\times 10^{-1}$	0.53	$1.65\times 10^{-2}$	33,500	45,000	1.00

**Table 3:** Directional response accuracy obtained in each evaluation run for the dense and fuzzy reward formulations.

Side	Approach 1 (Dense)	Approach 2 (Fuzzy)
R	(421/467)—90.15%	(407/458)—88.86%
R	(419/457)—91.68%	(393/437)—89.93%
R	(371/420)—88.33%	(386/428)—90.19%
R	(388/433)—89.61%	(404/441)—91.61%
R	(393/434)—90.55%	(400/439)—91.11%
L	(435/472)—92.16%	(455/477)—95.39%
L	(340/412)—82.52%	(431/469)—91.90%
L	(377/417)—90.41%	(350/398)—87.94%
L	(388/408)—95.10%	(371/402)—92.29%
L	(406/442)—91.86%	(380/420)—90.48%

**Side**: right side (R), left side (L).

**Table 4:** Task compliance rate obtained in each evaluation run for the dense and fuzzy reward formulations.

Side	Approach 1 (Dense)	Approach 2 (Fuzzy)
R	(370/490)—75.51%	(396/490)—80.82%
R	(380/490)—77.55%	(405/490)—82.65%
R	(394/490)—80.41%	(411/490)—83.87%
R	(414/490)—84.49%	(420/490)—85.71%
R	(408/490)—83.27%	(409/490)—83.47%
L	(263/490)—53.67%	(369/490)—75.31%
L	(295/490)—60.20%	(361/490)—73.67%
L	(316/490)—64.49%	(321/490)—65.51%
L	(381/490)—77.76%	(367/490)—74.90%
L	(360/490)—73.47%	(352/490)—71.84%

**Side**: right side (R), left side (L).

## Data Availability

The laparoscopic robot control application used in this study is openly available at https://github.com/ielguea/OSLO (accessed on 28 April 2026). The datasets generated and analyzed for human activity recognition, as well as the RL code developed for this work, are not publicly available because they are part of an ongoing study.

## Abbreviations

The following abbreviations are used in this manuscript:

APF	Artificial Potential Field
DDPG	Deep Deterministic Policy Gradient
DQN	Deep Q-Network
HRI	Human–Robot Interaction
MDP	Markov Decision Process
PER	Prioritized Experience Replay
PPO	Proximal Policy Optimization
RCM	Remote Center of Motion
RL	Reinforcement Learning
RULA	Rapid Upper Limb Assessment
SAC	Soft Actor–Critic
SEW	Shoulder–Elbow–Wrist
